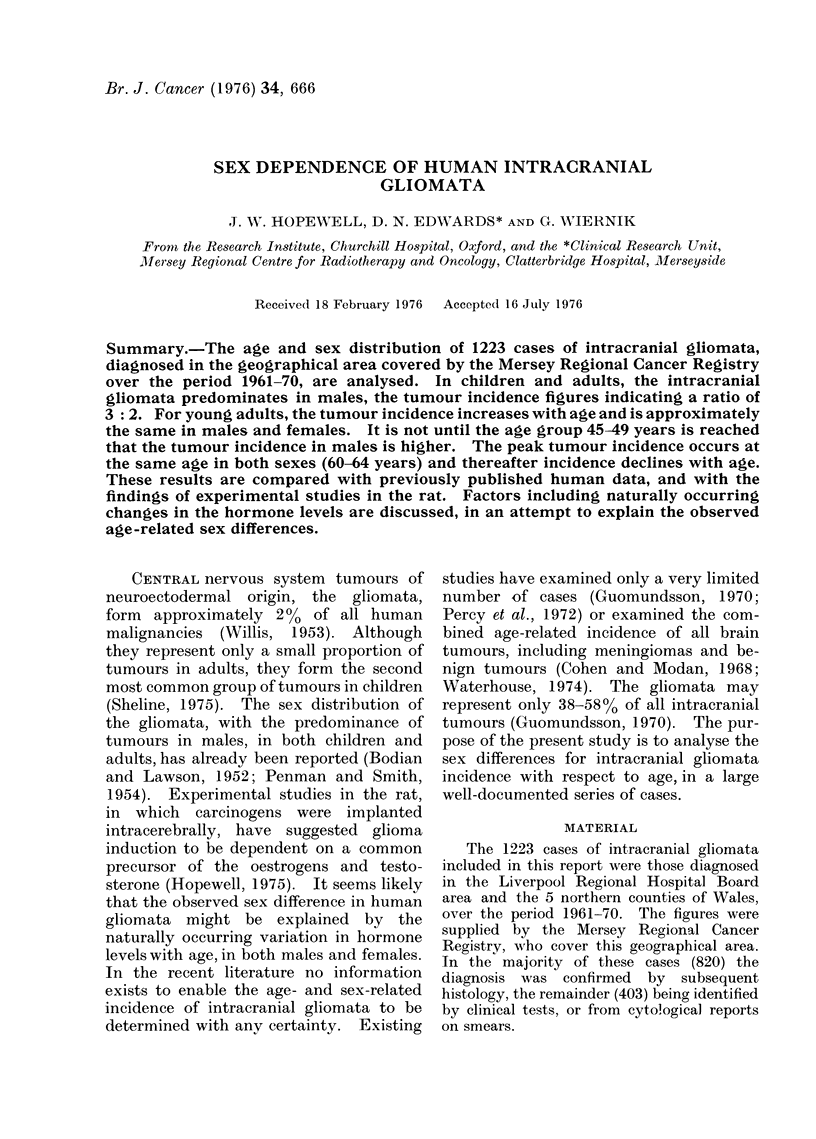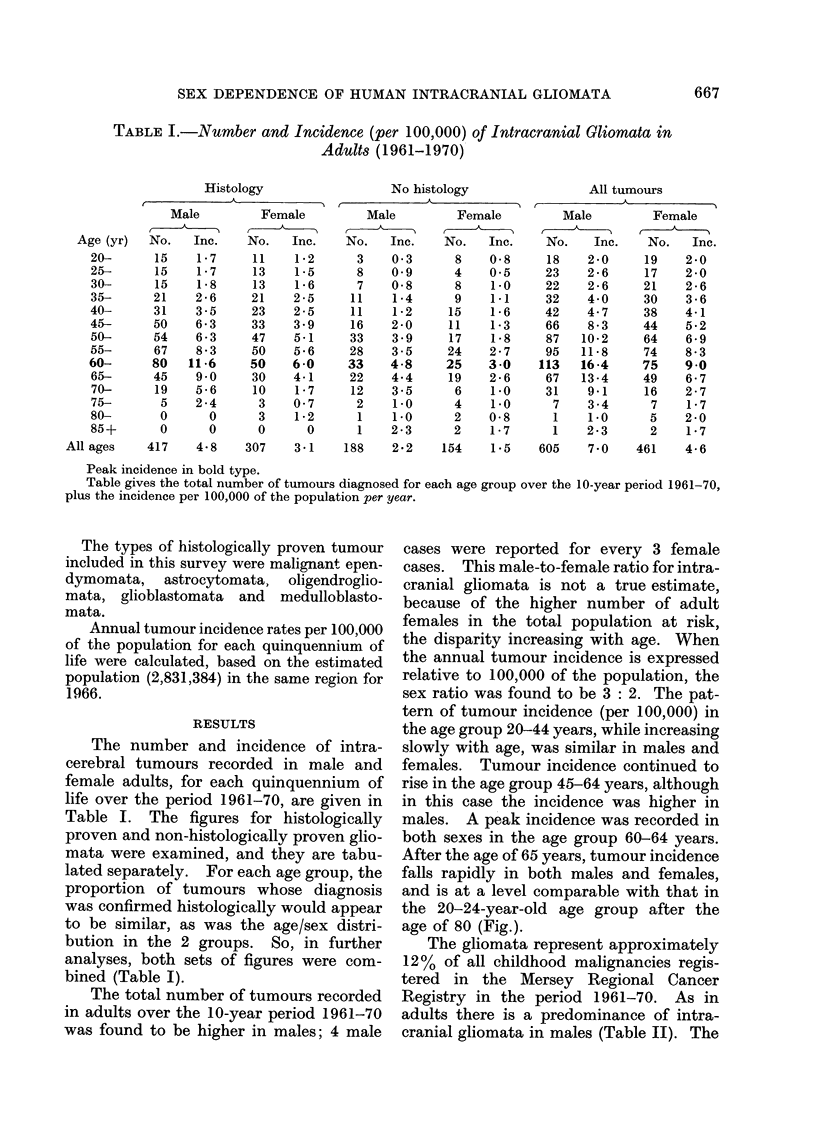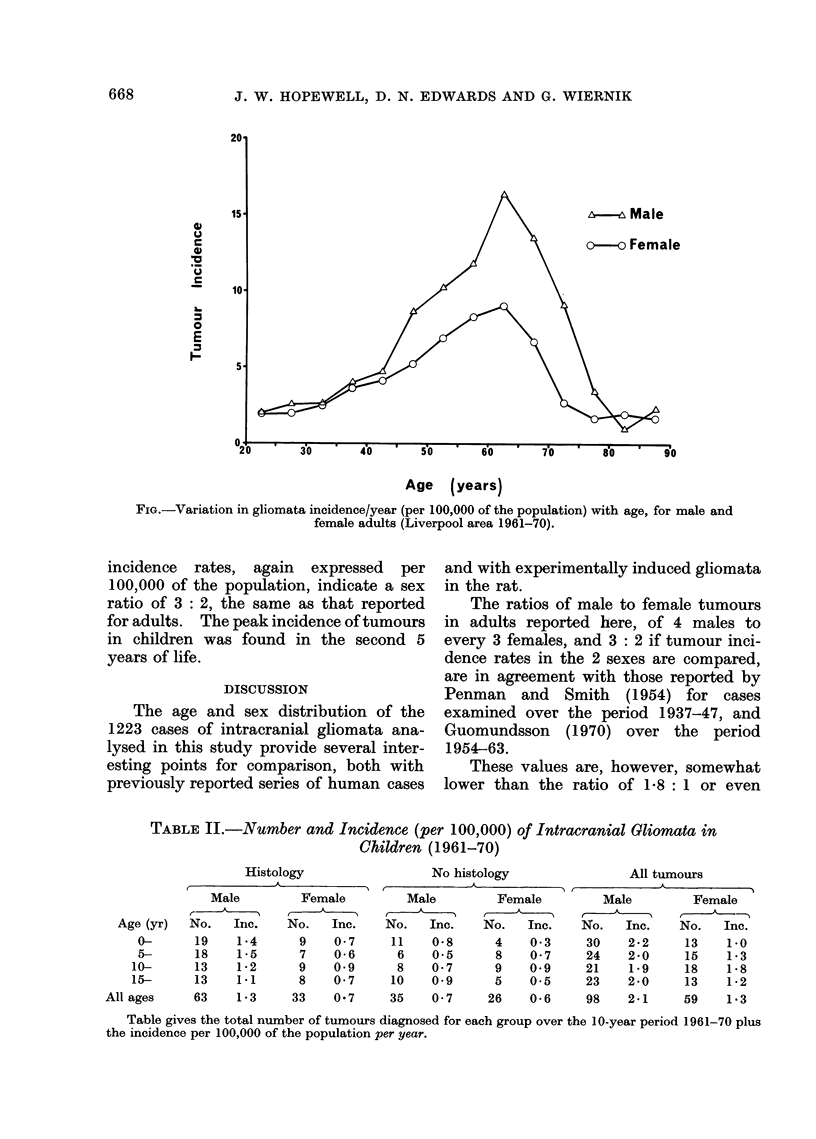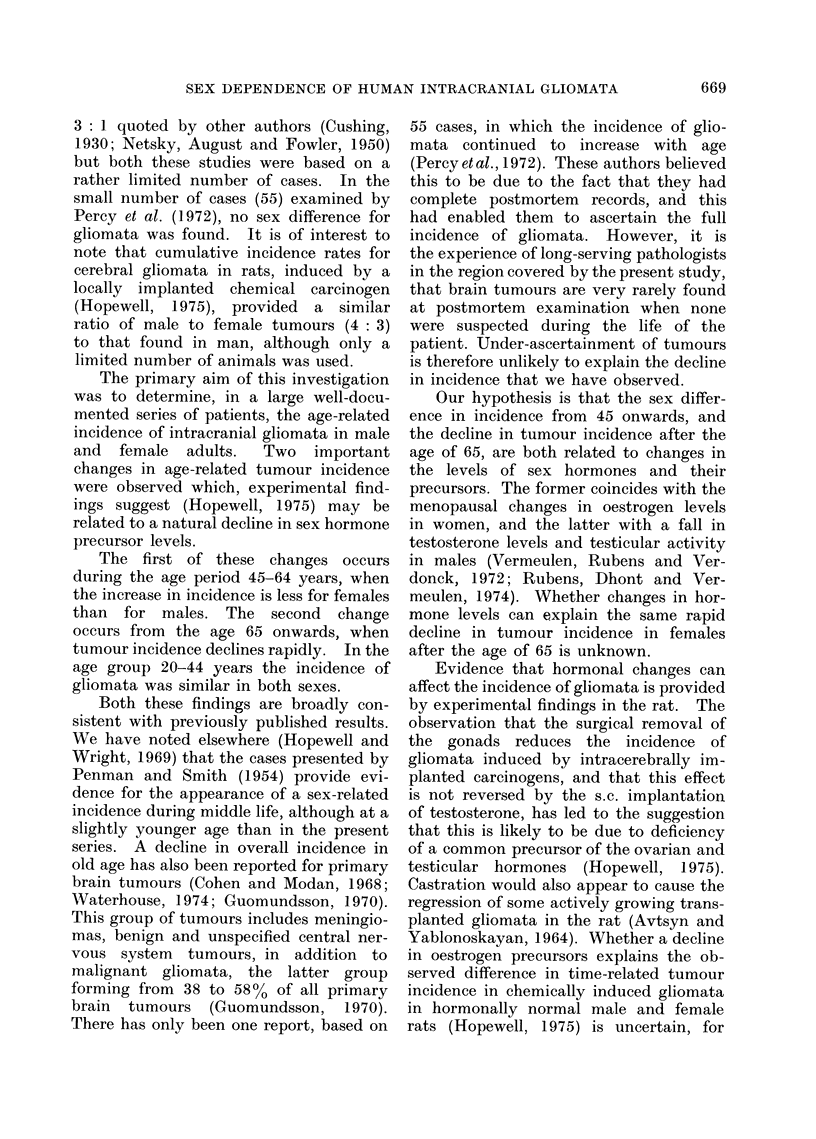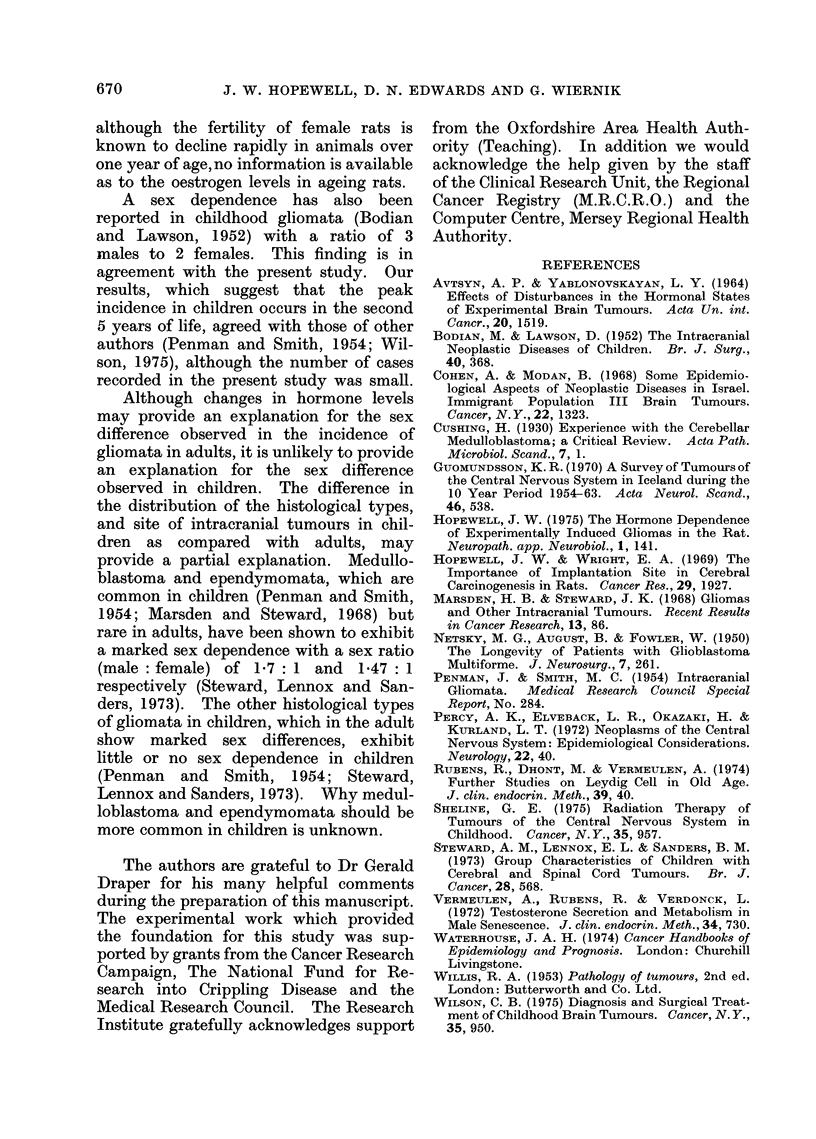# Sex dependence of human intracranial gliomata.

**DOI:** 10.1038/bjc.1976.230

**Published:** 1976-12

**Authors:** J. W. Hopewell, D. N. Edwards, G. Wiernik

## Abstract

The age and sex distribution of 1223 cases of intracranial gliomata, diagnosed in the geographical area covered by the Mersey Regional Cancer Registry over the period 1961-70, are analysed. In children and adults, the intracranial gliomata predominates in males, the tumour incidence figures indicating a ratio of 3 : 2. For young adults, the tumour incidence increases with age and is approximately the same in males and females. It is not until the age group 45-49 years is reached that the tumour incidence in males is higher. The peak tumour incidence occurs at the same age in both sexes (60-64 years) and thereafter incidence declines with age. These results are compared with previously published human data, and with the findings of experimental studies in the rat. Factors including naturally occurring changes in the hormone levels are discussed, in an attempt to explain the observed age-related sex differences.


*Br. J. Cancer* (1976) **34**, 666

### SEX DEPENDENCE OF HUMAN INTRACRANIAL GLIOMATA

J. W. HOPEWELL, D. N. EDWARDS* AND G. WIERNIK

*From the Research Institute, Churchill Hospital, Oxford, and the *Clinical Research Unit, Mersey Regional Centre for Radiotherapy and Oncology, Clatterbridge Hospital, Merseyside*

Received 18 February 1976 Accepted 16 July 1976

**Summary.—The age and sex distribution of 1223 cases of intracranial gliomata, diagnosed in the geographical area covered by the Mersey Regional Cancer Registry over the period 1961–70, are analysed. In children and adults, the intracranial gliomata predominates in males, the tumour incidence figures indicating a ratio of 3 : 2. For young adults, the tumour incidence increases with age and is approximately the same in males and females. It is not until the age group 45–49 years is reached that the tumour incidence in males is higher. The peak tumour incidence occurs at the same age in both sexes (60–64 years) and thereafter incidence declines with age. These results are compared with previously published human data, and with the findings of experimental studies in the rat. Factors including naturally occurring changes in the hormone levels are discussed, in an attempt to explain the observed age-related sex differences.**

CENTRAL nervous system tumours of neuroectodermal origin, the gliomata, form approximately 2% of all human malignancies (Willis, 1953). Although they represent only a small proportion of tumours in adults, they form the second most common group of tumours in children (Sheline, 1975). The sex distribution of the gliomata, with the predominance of tumours in males, in both children and adults, has already been reported (Bodian and Lawson, 1952; Penman and Smith, 1954). Experimental studies in the rat, in which carcinogens were implanted intracerebrally, have suggested glioma induction to be dependent on a common precursor of the oestrogens and testosterone (Hopewell, 1975). It seems likely that the observed sex difference in human gliomata might be explained by the naturally occurring variation in hormone levels with age, in both males and females. In the recent literature no information exists to enable the age- and sex-related incidence of intracranial gliomata to be determined with any certainty. Existing studies have examined only a very limited number of cases (Guomundsson, 1970; Percy *et al.*, 1972) or examined the combined age-related incidence of all brain tumours, including meningiomas and benign tumours (Cohen and Modan, 1968; Waterhouse, 1974). The gliomata may represent only 38–58% of all intracranial tumours (Guomundsson, 1970). The purpose of the present study is to analyse the sex differences for intracranial gliomata incidence with respect to age, in a large well-documented series of cases.

### MATERIAL

The 1223 cases of intracranial gliomata included in this report were those diagnosed in the Liverpool Regional Hospital Board area and the 5 northern counties of Wales, over the period 1961–70. The figures were supplied by the Mersey Regional Cancer Registry, who cover this geographical area. In the majority of these cases (820) the diagnosis was confirmed by subsequent histology, the remainder (403) being identified by clinical tests, or from cytological reports on smears.

TABLE I.—*Number and Incidence (per* 100,000) *of Intracranial Gliomata in Adults* (1961–1970)

| | Histology | | | | No histology | | | | All tumours | | | |
|---|---|---|---|---|---|---|---|---|---|---|---|---|
| | Male | | Female | | Male | | Female | | Male | | Female | |
| Age (yr) | No. | Inc. | No. | Inc. | No. | Inc. | No. | Inc. | No. | Inc. | No. | Inc. |
| 20– | 15 | 1·7 | 11 | 1·2 | 3 | 0·3 | 8 | 0·8 | 18 | 2·0 | 19 | 2·0 |
| 25– | 15 | 1·7 | 13 | 1·5 | 8 | 0·9 | 4 | 0·5 | 23 | 2·6 | 17 | 2·0 |
| 30– | 15 | 1·8 | 13 | 1·6 | 7 | 0·8 | 8 | 1·0 | 22 | 2·6 | 21 | 2·6 |
| 35– | 21 | 2·6 | 21 | 2·5 | 11 | 1·4 | 9 | 1·1 | 32 | 4·0 | 30 | 3·6 |
| 40– | 31 | 3·5 | 23 | 2·5 | 11 | 1·2 | 15 | 1·6 | 42 | 4·7 | 38 | 4·1 |
| 45– | 50 | 6·3 | 33 | 3·9 | 16 | 2·0 | 11 | 1·3 | 66 | 8·3 | 44 | 5·2 |
| 50– | 54 | 6·3 | 47 | 5·1 | 33 | 3·9 | 17 | 1·8 | 87 | 10·2 | 64 | 6·9 |
| 55– | 67 | 8·3 | 50 | 5·6 | 28 | 3·5 | 24 | 2·7 | 95 | 11·8 | 74 | 8·3 |
| **60–** | **80** | **11·6** | **50** | **6·0** | **33** | **4·8** | **25** | **3·0** | **113** | **16·4** | **75** | **9·0** |
| 65– | 45 | 9·0 | 30 | 4·1 | 22 | 4·4 | 19 | 2·6 | 67 | 13·4 | 49 | 6·7 |
| 70– | 19 | 5·6 | 10 | 1·7 | 12 | 3·5 | 6 | 1·0 | 31 | 9·1 | 16 | 2·7 |
| 75– | 5 | 2·4 | 3 | 0·7 | 2 | 1·0 | 4 | 1·0 | 7 | 3·4 | 7 | 1·7 |
| 80– | 0 | 0 | 3 | 1·2 | 1 | 1·0 | 2 | 0·8 | 1 | 1·0 | 5 | 2·0 |
| 85+ | 0 | 0 | 0 | 0 | 1 | 2·3 | 2 | 1·7 | 1 | 2·3 | 2 | 1·7 |
| All ages | 417 | 4·8 | 307 | 3·1 | 188 | 2·2 | 154 | 1·5 | 605 | 7·0 | 461 | 4·6 |

Peak incidence in bold type.
Table gives the total number of tumours diagnosed for each age group over the 10-year period 1961–70, plus the incidence per 100,000 of the population *per year*.

The types of histologically proven tumour included in this survey were malignant ependymomata, astrocytomata, oligendrogliomata, glioblastomata and medulloblastomata.

Annual tumour incidence rates per 100,000 of the population for each quinquennium of life were calculated, based on the estimated population (2,831,384) in the same region for 1966.

### RESULTS

The number and incidence of intracerebral tumours recorded in male and female adults, for each quinquennium of life over the period 1961–70, are given in Table I. The figures for histologically proven and non-histologically proven gliomata were examined, and they are tabulated separately. For each age group, the proportion of tumours whose diagnosis was confirmed histologically would appear to be similar, as was the age/sex distribution in the 2 groups. So, in further analyses, both sets of figures were combined (Table I).

The total number of tumours recorded in adults over the 10-year period 1961–70 was found to be higher in males; 4 male cases were reported for every 3 female cases. This male-to-female ratio for intracranial gliomata is not a true estimate, because of the higher number of adult females in the total population at risk, the disparity increasing with age. When the annual tumour incidence is expressed relative to 100,000 of the population, the sex ratio was found to be 3 : 2. The pattern of tumour incidence (per 100,000) in the age group 20–44 years, while increasing slowly with age, was similar in males and females. Tumour incidence continued to rise in the age group 45–64 years, although in this case the incidence was higher in males. A peak incidence was recorded in both sexes in the age group 60–64 years. After the age of 65 years, tumour incidence falls rapidly in both males and females, and is at a level comparable with that in the 20–24-year-old age group after the age of 80 (Fig.).

The gliomata represent approximately 12% of all childhood malignancies registered in the Mersey Regional Cancer Registry in the period 1961–70. As in adults there is a predominance of intracranial gliomata in males (Table II). The

FIG.—Variation in gliomata incidence/year (per 100,000 of the population) with age, for male and female adults (Liverpool area 1961–70).

incidence rates, again expressed per 100,000 of the population, indicate a sex ratio of 3 : 2, the same as that reported for adults. The peak incidence of tumours in children was found in the second 5 years of life.

DISCUSSION

The age and sex distribution of the 1223 cases of intracranial gliomata analysed in this study provide several interesting points for comparison, both with previously reported series of human cases and with experimentally induced gliomata in the rat.

The ratios of male to female tumours in adults reported here, of 4 males to every 3 females, and 3 : 2 if tumour incidence rates in the 2 sexes are compared, are in agreement with those reported by Penman and Smith (1954) for cases examined over the period 1937–47, and Guomundsson (1970) over the period 1954–63.

These values are, however, somewhat lower than the ratio of 1·8 : 1 or even

TABLE II.—*Number and Incidence* (*per* 100,000) *of Intracranial Gliomata in Children* (1961–70)

| | Histology | | | | No histology | | | | All tumours | | | |
|---|---|---|---|---|---|---|---|---|---|---|---|---|
| | Male | | Female | | Male | | Female | | Male | | Female | |
| Age (yr) | No. | Inc. | No. | Inc. | No. | Inc. | No. | Inc. | No. | Inc. | No. | Inc. |
| 0– | 19 | 1·4 | 9 | 0·7 | 11 | 0·8 | 4 | 0·3 | 30 | 2·2 | 13 | 1·0 |
| 5– | 18 | 1·5 | 7 | 0·6 | 6 | 0·5 | 8 | 0·7 | 24 | 2·0 | 15 | 1·3 |
| 10– | 13 | 1·2 | 9 | 0·9 | 8 | 0·7 | 9 | 0·9 | 21 | 1·9 | 18 | 1·8 |
| 15– | 13 | 1·1 | 8 | 0·7 | 10 | 0·9 | 5 | 0·5 | 23 | 2·0 | 13 | 1·2 |
| All ages | 63 | 1·3 | 33 | 0·7 | 35 | 0·7 | 26 | 0·6 | 98 | 2·1 | 59 | 1·3 |

Table gives the total number of tumours diagnosed for each group over the 10-year period 1961–70 plus the incidence per 100,000 of the population *per year*.

3 : 1 quoted by other authors (Cushing, 1930; Netsky, August and Fowler, 1950) but both these studies were based on a rather limited number of cases. In the small number of cases (55) examined by Percy *et al.* (1972), no sex difference for gliomata was found. It is of interest to note that cumulative incidence rates for cerebral gliomata in rats, induced by a locally implanted chemical carcinogen (Hopewell, 1975), provided a similar ratio of male to female tumours (4 : 3) to that found in man, although only a limited number of animals was used.

The primary aim of this investigation was to determine, in a large well-documented series of patients, the age-related incidence of intracranial gliomata in male and female adults. Two important changes in age-related tumour incidence were observed which, experimental findings suggest (Hopewell, 1975) may be related to a natural decline in sex hormone precursor levels.

The first of these changes occurs during the age period 45–64 years, when the increase in incidence is less for females than for males. The second change occurs from the age 65 onwards, when tumour incidence declines rapidly. In the age group 20–44 years the incidence of gliomata was similar in both sexes.

Both these findings are broadly consistent with previously published results. We have noted elsewhere (Hopewell and Wright, 1969) that the cases presented by Penman and Smith (1954) provide evidence for the appearance of a sex-related incidence during middle life, although at a slightly younger age than in the present series. A decline in overall incidence in old age has also been reported for primary brain tumours (Cohen and Modan, 1968; Waterhouse, 1974; Guomundsson, 1970). This group of tumours includes meningiomas, benign and unspecified central nervous system tumours, in addition to malignant gliomata, the latter group forming from 38 to 58% of all primary brain tumours (Guomundsson, 1970). There has only been one report, based on 55 cases, in which the incidence of gliomata continued to increase with age (Percy *et al.*, 1972). These authors believed this to be due to the fact that they had complete postmortem records, and this had enabled them to ascertain the full incidence of gliomata. However, it is the experience of long-serving pathologists in the region covered by the present study, that brain tumours are very rarely found at postmortem examination when none were suspected during the life of the patient. Under-ascertainment of tumours is therefore unlikely to explain the decline in incidence that we have observed.

Our hypothesis is that the sex difference in incidence from 45 onwards, and the decline in tumour incidence after the age of 65, are both related to changes in the levels of sex hormones and their precursors. The former coincides with the menopausal changes in oestrogen levels in women, and the latter with a fall in testosterone levels and testicular activity in males (Vermeulen, Rubens and Verdonck, 1972; Rubens, Dhont and Vermeulen, 1974). Whether changes in hormone levels can explain the same rapid decline in tumour incidence in females after the age of 65 is unknown.

Evidence that hormonal changes can affect the incidence of gliomata is provided by experimental findings in the rat. The observation that the surgical removal of the gonads reduces the incidence of gliomata induced by intracerebrally implanted carcinogens, and that this effect is not reversed by the s.c. implantation of testosterone, has led to the suggestion that this is likely to be due to deficiency of a common precursor of the ovarian and testicular hormones (Hopewell, 1975). Castration would also appear to cause the regression of some actively growing transplanted gliomata in the rat (Avtsyn and Yablonoskayan, 1964). Whether a decline in oestrogen precursors explains the observed difference in time-related tumour incidence in chemically induced gliomata in hormonally normal male and female rats (Hopewell, 1975) is uncertain, for although the fertility of female rats is known to decline rapidly in animals over one year of age, no information is available as to the oestrogen levels in ageing rats.

A sex dependence has also been reported in childhood gliomata (Bodian and Lawson, 1952) with a ratio of 3 males to 2 females. This finding is in agreement with the present study. Our results, which suggest that the peak incidence in children occurs in the second 5 years of life, agreed with those of other authors (Penman and Smith, 1954; Wilson, 1975), although the number of cases recorded in the present study was small.

Although changes in hormone levels may provide an explanation for the sex difference observed in the incidence of gliomata in adults, it is unlikely to provide an explanation for the sex difference observed in children. The difference in the distribution of the histological types, and site of intracranial tumours in children as compared with adults, may provide a partial explanation. Medulloblastoma and ependymomata, which are common in children (Penman and Smith, 1954; Marsden and Steward, 1968) but rare in adults, have been shown to exhibit a marked sex dependence with a sex ratio (male : female) of 1·7 : 1 and 1·47 : 1 respectively (Steward, Lennox and Sanders, 1973). The other histological types of gliomata in children, which in the adult show marked sex differences, exhibit little or no sex dependence in children (Penman and Smith, 1954; Steward, Lennox and Sanders, 1973). Why medulloblastoma and ependymomata should be more common in children is unknown.

The authors are grateful to Dr Gerald Draper for his many helpful comments during the preparation of this manuscript. The experimental work which provided the foundation for this study was supported by grants from the Cancer Research Campaign, The National Fund for Research into Crippling Disease and the Medical Research Council. The Research Institute gratefully acknowledges support from the Oxfordshire Area Health Authority (Teaching). In addition we would acknowledge the help given by the staff of the Clinical Research Unit, the Regional Cancer Registry (M.R.C.R.O.) and the Computer Centre, Mersey Regional Health Authority.

### REFERENCES

Avtsyn, A. P. & Yablonovskayan, L. Y. (1964) Effects of Disturbances in the Hormonal States of Experimental Brain Tumours. *Acta Un. int. Cancr.*, **20**, 1519.

Bodian, M. & Lawson, D. (1952) The Intracranial Neoplastic Diseases of Children. *Br. J. Surg.*, **40**, 368.

Cohen, A. & Modan, B. (1968) Some Epidemiological Aspects of Neoplastic Diseases in Israel. Immigrant Population III Brain Tumours. *Cancer, N.Y.*, **22**, 1323.

Cushing, H. (1930) Experience with the Cerebellar Medulloblastoma; a Critical Review. *Acta Path. Microbiol. Scand.*, **7**, 1.

Guomundsson, K. R. (1970) A Survey of Tumours of the Central Nervous System in Iceland during the 10 Year Period 1954–63. *Acta Neurol. Scand.*, **46**, 538.

Hopewell, J. W. (1975) The Hormone Dependence of Experimentally Induced Gliomas in the Rat. *Neuropath. app. Neurobiol.*, **1**, 141.

Hopewell, J. W. & Wright, E. A. (1969) The Importance of Implantation Site in Cerebral Carcinogenesis in Rats. *Cancer Res.*, **29**, 1927.

Marsden, H. B. & Steward, J. K. (1968) Gliomas and Other Intracranial Tumours. *Recent Results in Cancer Research*, **13**, 86.

Netsky, M. G., August, B. & Fowler, W. (1950) The Longevity of Patients with Glioblastoma Multiforme. *J. Neurosurg.*, **7**, 261.

Penman, J. & Smith, M. C. (1954) Intracranial Gliomata. *Medical Research Council Special Report*, No. 284.

Percy, A. K., Elveback, L. R., Okazaki, H. & Kurland, L. T. (1972) Neoplasms of the Central Nervous System: Epidemiological Considerations. *Neurology*, **22**, 40.

Rubens, R., Dhont, M. & Vermeulen, A. (1974) Further Studies on Leydig Cell in Old Age. *J. clin. endocrin. Meth.*, **39**, 40.

Sheline, G. E. (1975) Radiation Therapy of Tumours of the Central Nervous System in Childhood. *Cancer, N.Y.*, **35**, 957.

Steward, A. M., Lennox, E. L. & Sanders, B. M. (1973) Group Characteristics of Children with Cerebral and Spinal Cord Tumours. *Br. J. Cancer*, **28**, 568.

Vermeulen, A., Rubens, R. & Verdonck, L. (1972) Testosterone Secretion and Metabolism in Male Senescence. *J. clin. endocrin. Meth.*, **34**, 730.

Waterhouse, J. A. H. (1974) *Cancer Handbooks of Epidemiology and Prognosis.* London: Churchill Livingstone.

Willis, R. A. (1953) *Pathology of tumours*, 2nd ed. London: Butterworth and Co. Ltd.

Wilson, C. B. (1975) Diagnosis and Surgical Treatment of Childhood Brain Tumours. *Cancer, N.Y.*, **35**, 950.